# Prevalence and Health-Adjusted Life Expectancy Among Older Adults With Hypertension in Chinese Rural Areas

**DOI:** 10.3389/fpubh.2022.802195

**Published:** 2022-03-01

**Authors:** Ze Hu, Xiaotian Liu, Wei Liao, Ning Kang, Lixia Ma, Zhenxing Mao, Jian Hou, Wenqian Huo, Yuqian Li, Chongjian Wang

**Affiliations:** ^1^Department of Epidemiology and Biostatistics, College of Public Health, Zhengzhou University, Zhengzhou, China; ^2^Department of Applied Statistics, School of Statistics and Big Data, Henan University of Economics and Law, Zhengzhou, China; ^3^Department of Clinical Pharmacology, School of Pharmaceutical Science, Zhengzhou University, Zhengzhou, China

**Keywords:** hypertension, prevalence, the elderly, rural regions, health-adjusted life expectancy

## Abstract

**Background:**

The objectives of the present study were to explore the epidemiological characteristics of hypertension among rural older adults in resource-limited regions, and then evaluate the loss of health-adjusted life expectancy due to hypertension.

**Methods:**

Participants aged between 60 and 79 years were enrolled from Henan rural cohort study. The prevalence, awareness, treatment, and control of hypertension were detailed across subgroups. Variances within subgroups were identified *via* Student's t tests or one-way ANOVA for continuous variables and chi-squared tests for categorical ones, and logistic regression model was employed to detect the potential influencing factors. The health-adjusted life expectancy was calculated by the Sullivan method with EuroqOL-5D data.

**Results:**

Among 16,785 participants, 7,472 (44.52%) were attacked by hypertension, 4,858 (65.02%) had been already aware of their condition, 4,009 (53.65%) were taking antihypertensive medication for treatment, while only 1,478 (19.78%) had their hypertension controlled. The prevalence of hypertension was significantly higher among women than men and it increased with age for both genders. For the older ones aged 60 years, the life expectancy was 22.0872 years and the health-adjusted life expectancy was 15.5578 and 15.9418 for those with or without hypertension, respectively. Namely, in this particular age group, subjects without hypertension could gain 0.3840 years of health-adjusted life expectancy.

**Conclusion:**

The prevalence of hypertension was relatively high while the awareness, treatment, and control were fairly low. The health-adjusted life expectancy of older adults in resource-limited areas could increase from the reduction of hypertension. There is an urgent need for strategies pertaining to the prevention and treatment of hypertension.

**Clinical Trial Registration:**

The Henan Rural Cohort Study has been registered at the Chinese Clinical Trial Register (Registration number: ChiCTR-OOC-15006699). Date of registration: 06 July, 2015. http://www.chictr.org.cn/showproj.aspx?proj=11375.

## Introduction

Hypertension, characterized as blood vessels having persistently raised pressure (1), was a well-known risk factor leading to cardiovascular diseases (CVDs), and has contributed to the majority of premature death globally (2). It has been reported that half of CVDs were caused by elevated blood pressure (BP), and more than 1.5 billion people are suffering from hypertension nowadays (3).

As the most populous country, China has been having soaring cases of hypertensives over the decades (4). With the rapid development of economy and a huge transition in dietary and life habits in China, the prevalence of hypertension has increased over the decades (5). It is worth mentioning that blood pressure increases with age (6), therefore, old population are of greater risk of hypertension. Additionally, a national survey published recently suggested that the prevalence of hypertension was higher in rural areas than urban ones in some developed areas in our country (7). The acceleration of aging and urbanization plays an important role in this phenomenon as well (8). Therefore, old population aged over 60 living in rural areas was a noteworthy crowd to which more attention should be paid to.

Over decades, substantial studies had presented the epidemiological characteristics and influencing factors of hypertension at home and abroad (4, 9, 10). However, researches covering a large sample among rural old population in central China was still limited. Hence, this large sample crosssectional study which involved the levels of blood pressure in conjunction with prevalence, awareness, treatment, and control of hypertension was conducted to provide the latest evidence on the current status of hypertension among older population in central rural China. Moreover, the present study also calculated life expectancy (LE) and health-adjusted life expectancy (HALE) in people with or without hypertension to intuitively illustrate the burden caused by hypertension in this population.

## Materials and Methods

### Study Population

Data was from the Henan Rural cohort and details of that were published elsewhere (11). Briefly, participants in this study were recruited from the Henan rural cohort, the baseline of which was launched from July 2015 to September 2017 and follow-up survey of which are still running. With a high response rate (93.7%), the Henan Rural Cohort Study containing 39,259 participants was carried out in five rural areas (Suiping, Yuzhou, Xinixiang, Tongxu, and Yima) of Henan province through a multistage, stratified cluster sampling method. Participants were excluded if they: (1) were under the age of 60 (*n* = 22,206); (2) were diagnosed with cancer (*n* = 174); (3) had serious renal disease (*n* = 10); (4) were diagnosed with gestational hypertension previously (*n* = 60); (5) did not have information on data of blood pressure (*n* = 20) and taking antihypertensive medications during previous 2 weeks (*n* = 4). Ultimately, 16,785 subjects (7,370 men and 9,415 women) were included in the present study. Among 16,785 study participants, 9,920 individuals finished the EQ-5D-5L questionnaire and were included in the analysis of health-adjusted life expectancy. A flow chart for the inclusion and exclusion of participants are displayed in the Supplementary Appendix.

The study was approved by Zhengzhou University Life Science Ethics Committee (Code: [2015] MEC (S128)) and was conducted following the principles of the Declaration of Helsinki. Additionally, written informed consent was obtained from each participant.

### Data Collection

A standard questionnaire containing information on general demographic characteristics, lifestyle characters, personal, and family history of diseases was employed by well-trained investigators during face-to-face interviews. Body mass index (BMI), as an index of general obesity, was calculated as weight (kg) divided by the average of height readings squared (m^2^) and further categorized as underweight (BMI <18.5 kg/m^2^), normal (18.5 kg/m^2^ ≤ BMI <24 kg/m^2^), overweight (24 kg/m^2^ ≤ BMI <28 kg/m^2^), or obese (BMI ≥ 28 kg/m^2^). The subjects' height and weight were measured in light clothes and without shoes following standard protocols.

Subjects were divided into four age groups: 60–64 years, 65–69 years, 70–74 years, and >75 years. Education levels were classified into three categories: illiterate, primary school, and middle school or above. Smoking and drinking status were categorized as never, former, and current. Taking vegetable and fruit more than 500 g per day was defined as more vegetable and fruit intake, and a high-fat diet was defined if someone on an average eats meat of livestock and poultry of more than 75 g per day (12). Physical activity was categorized as low, moderate, and high according to the International Physical Activity Questionnaire (IPAQ) (13). The IPAQ and the cutoffs used for low, moderate, and high activity have been provided in the Supplementary Appendix.

### Measurement of Blood Pressure

According to the JNC 7 Report in 2003 measurements (14), participants were asked to rest for at least 5 min before measurements. With an electronic sphygmomanometer (HEM-770AFuzzy, Omron, Japan), the resting blood pressure (BP) was measured three times with 30-s intervals between measurements, and then the average reading was applied in further analyses.

### Outcome Definition

Subjects would be diagnosed with prehypertension if their BP meet the following criteria (15): 120 mmHg ≤ SBP <140 mmHg and/or 80 mmHg ≤ DBP <90 mmHg. Hypertension was defined as the following standards (16): (1) SBP ≥140 mmHg and/or DBP ≥ 90 mmHg; (2) self-reported hypertension diagnosed by physicians previously and took antihypertensive medications in the past 2 weeks. The criteria of diabetes and dyslipidemia are described in detail in the Supplementary Appendix.

### Assessment of Health Adjusted Life Expectancy

With information on population in this study and death data from the 2017 China Cause-of-death Surveillance Data set, life expectancy (LE) was measured. Health adjusted life expectancy (HALE) of participants with or without hypertension was further calculated using the Sullivan method (17) with life expectancy (LE) and EuroqOL-5D (EQ-5D) data. The steps of HALE calculation are displayed in Supplementary Appendix.

### Statistical Analysis

Continue variables were expressed as means ± SDs, and intergroup differences were detected *via* Student's *t*-test or one-way ANOVA, while categorical ones were presented as frequencies (percentages) and chi-squared tests were applied to compare variances between groups. In this survey, a full-adjusted logistic regression model was employed to analyze the connection of potential influencing factors with the prevalence, awareness, treatment, and control of hypertension. Age-standardized prevalence, awareness, treatment, and control of hypertension were computed according to data from the 2010 Chinese census (18). *P* < 0.05 (two-sided test) was considered to be statistically significant in the present study. SPSS version 21.0 was used to carry out statistical analysis.

## Results

### Demographic Characteristics

As presented in Table 1, among 16,785 participants covered in this study, 5,048 (30.07%) individuals were suffering from prehypertension and 7,472 (44.52%) were diagnosed with hypertension. Participants with prehypertension and hypertension were more inclined to be older, women, single/widowed/separated/divorced, lower educated, never drinkers and never smokers, and more likely to have lower monthly income, higher body mass index (BMI), lower physical activity, higher family history of hypertension, and higher prevalence of diabetes or dyslipidemia (all *P* < 0.05). However, they were less likely to have high-fat diet and more vegetable and fruit intake (*P* < 0.05). The self-reported damage of participants (no, slight, moderate, severe, or extreme problems) based on the EQ-5D-5L questionnaire are summarized in Supplementary Table 1.

**Table 1:** Demographic of study participants according to blood pressure status.

**Variable**	**Normotension**	**Prehypertension**	**Hypertension**	***P***
	**(*N* = 4,265)**	**(*N* = 5,048)**	**(*N* = 7,472)**	
Age (year), mean ± SD	65.97 ± 4.58	66.50 ± 4.80	67.32 ± 4.97	<0.001
Age group (year)	1,941 (45.51)	2,075 (41.11)	2,583 (34.57)	<0.001
60~64	1,379 (32.33)	1,658 (32.84)	2,455 (32.86)	
65~69	694 (16.27)	893 (17.69)	1,640 (21.95)	
70~74	251 (5.89)	422 (8.36)	794 (10.63)	
75~				
Gender (men), *n* (%)	2,151 (50.43)	2,303 (45.62)	2,916 (39.03)	<0.001
Marital status, *n* (%)				<0.001
Married/cohabiting	3,619 (84.85)	4,217 (83.54)	6,056 (81.05)	
Widowed/single/divorced/separation	646 (15.15)	831 (16.46)	1,416 (18.95)	
Education^*^, *n* (%)				<0.001
Illiterate	1,287 (30.18)	1,558 (30.86)	2,404 (32.17)	
Primary school	1,514 (35.50)	1,791 (35.48)	2,920 (39.08)	
Middle school and above	1,464 (34.33)	1,699 (33.66)	2,148 (28.75)	
Per capita monthly income (RMB), *n* (%)				0.026
<500	1,971 (46.21)	2,313 (45.82)	3,554 (47.56)	
500~1000	1,304 (30.57)	1,565 (31.00)	2,340 (31.32)	
>1000	990 (23.21)	1,170 (23.18)	1,578 (21.12)	
Body mass index (kg/m^2^), mean ± SD	22.96 ± 3.18	24.29 ± 3.39	25.49 ± 3.61	<0.001
High fat diet, *n* (%)	639 (14.98)	697 (13.81)	802 (10.73)	<0.001
More vegetable and fruit intake, *n* (%)	1,899 (44.53)	1,968 (38.99)	2,470 (33.06)	<0.001
Smoking, *n* (%)				<0.001
Never	2,707 (63.47)	3,449 (68.32)	5,560 (74.41)	
Former	452 (10.60)	582 (11.53)	913 (12.22)	
Current	1,106 (25.93)	1,017 (20.15)	999 (13.37)	
Drinking, *n* (%)				<0.001
Never	3,283 (76.98)	3,938 (78.01)	6,033 (80.74)	
Former	320 (7.50)	305 (6.04)	481 (6.44)	
Current	662 (15.52)	805 (15.95)	958 (12.82)	
Physical activity, *n* (%)				<0.001
Low	1,313 (30.79)	1,717 (34.01)	3,173 (42.47)	
Moderate	1,621 (38.01)	1,823 (36.11)	2,325 (31.12)	
High	1331 (31.21)	1508 (29.87)	1974 (26.42)	
Family history of hypertension, *n* (%)	265 (6.21)	349 (6.91)	1,465 (19.61)	<0.001
Dyslipidemia, *n* (%)	1,224 (28.74)	1,770 (35.11)	3,426 (45.94)	<0.001
Diabetes, *n* (%)	328 (7.71)	568 (11.27)	1,234 (16.55)	<0.001

* *In our study, primary school represents six schooling years, and middle school represents three more years based on primary school*.

### Prevalence of Prehypertension and Hypertension

**Figure 1:**
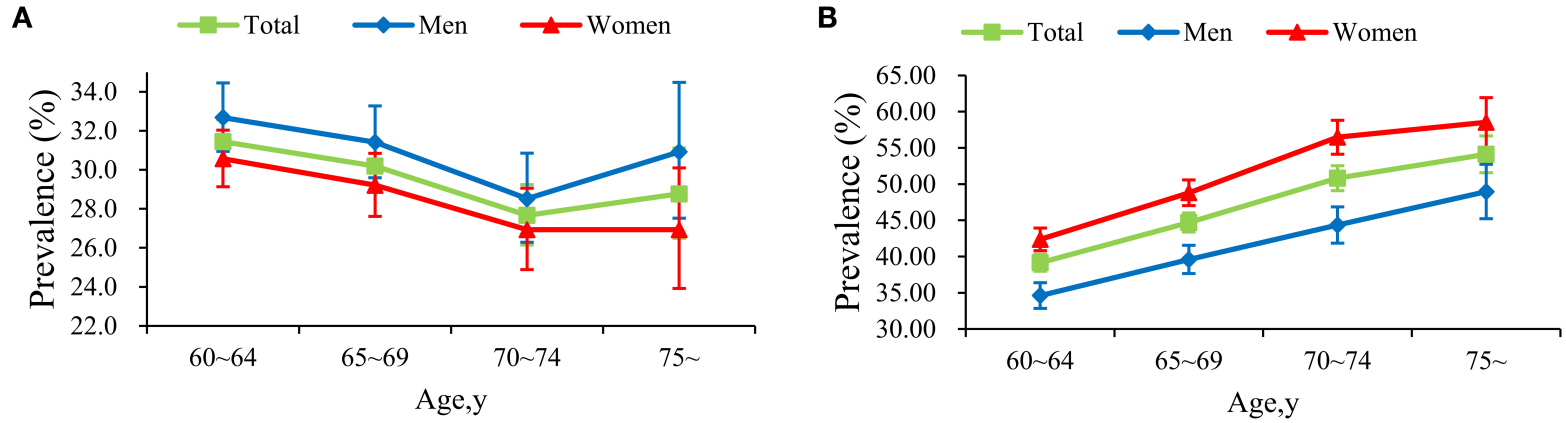
Prevalence of prehypertension **(A)** and hypertension **(B)** among different subgroups.

Overall, the prevalence of prehypertension and hypertension was 30.07 and 44.52%, and age-standardized prevalence was 29.83 and 45.36%, respectively. The prevalence of prehypertension with age was distributed as U-shape, touching the bottom at the age of 70–74 for both genders. Figure 1 also sheds light on that the prevalence of hypertension increased with age for both genders. Hypertension was more prevalent among women (48.39 vs. 39.57%, *P* < 0.001), while prehypertension was more common among men (31.25 vs. 29.16%, *P* = 0.003). In addition, the prevalence of hypertension increased with age and BMI (both *P*_*trend*_ <0.001). More details toward the prevalence of prehypertension and hypertension were displayed in Table 2.

**Table 2:** The prevalence of prehypertension and hypertension of total participants.

**Variable**	**Prehypertension, *n* (%)**	***P***	**Hypertension, *n* (%)**	***P***
Gender		0.003		<0.001
Men	2,303 (31.25)		2,916 (39.57)	
Women	2,745 (29.16)		4,556 (48.39)	
Marital status		0.082		<0.001
Married/cohabiting	4,217 (30.36)		6,056 (43.59)	
Widowed/single/divorced/separation	831 (28.72)		1,416 (48.95)	
Education^*^		0.001		<0.001
Illiterate	1,558 (29.68)		2,404 (45.80)	
Primary school	1,791 (28.77)		2,920 (46.91)	
Middle school and above	1,699 (31.99)		2,148 (40.44)	
Per capita monthly income (RMB)		0.145		0.004
<500	2,313 (29.51)		3,554 (45.34)	
500~1000	1,565 (30.04)		2,340 (44.92)	
>1000	1,170 (31.30)		1,578 (42.22)	
Body mass index (kg/m2)		<0.001		<0.001
Underweight	150 (26.41)		122 (21.48)	
Normal	2,289 (31.70)		2,485 (34.42)	
Overweight	1,918 (30.37)		3,149 (49.87)	
Obese	676 (25.93)		1,671 (64.10)	
High fat diet	697 (32.60)	0.006	802 (37.51)	<0.001
More vegetable and fruit intake	1,968 (31.06)	0.031	2,470 (38.98)	<0.001
Smoking		0.003		<0.001
Never	3,449 (29.44)		5,560 (47.46)	
Former	582 (29.89)		913 (46.89)	
Current	1,017 (32.58)		999 (32.00)	
Drinking		<0.001		<0.001
Never	3,938 (29.71)		6,033 (45.52)	
Former	305 (27.58)		481 (43.49)	
Current	805 (33.20)		958 (39.51)	
Physical activity		<0.001		<0.001
Low	1,717 (27.68)		3,173 (51.15)	
Moderate	1,823 (31.60)		2,325 (40.30)	
High	1,508 (31.33)		1,974 (41.01)	
Family history of hypertension	349 (16.79)	<0.001	1,465 (70.47)	<0.001
Dyslipidemia	1,770 (27.57)	<0.001	3,426 (53.36)	<0.001
Diabetes	568 (26.67)	<0.001	1,234 (57.93)	<0.001

* *In our study, primary school represents six schooling years and middle school represents three more years based on primary school, and we added the schooling years in the revised manuscript*.

### Awareness, Treatment, and Control of Hypertension

**Figure 2:**
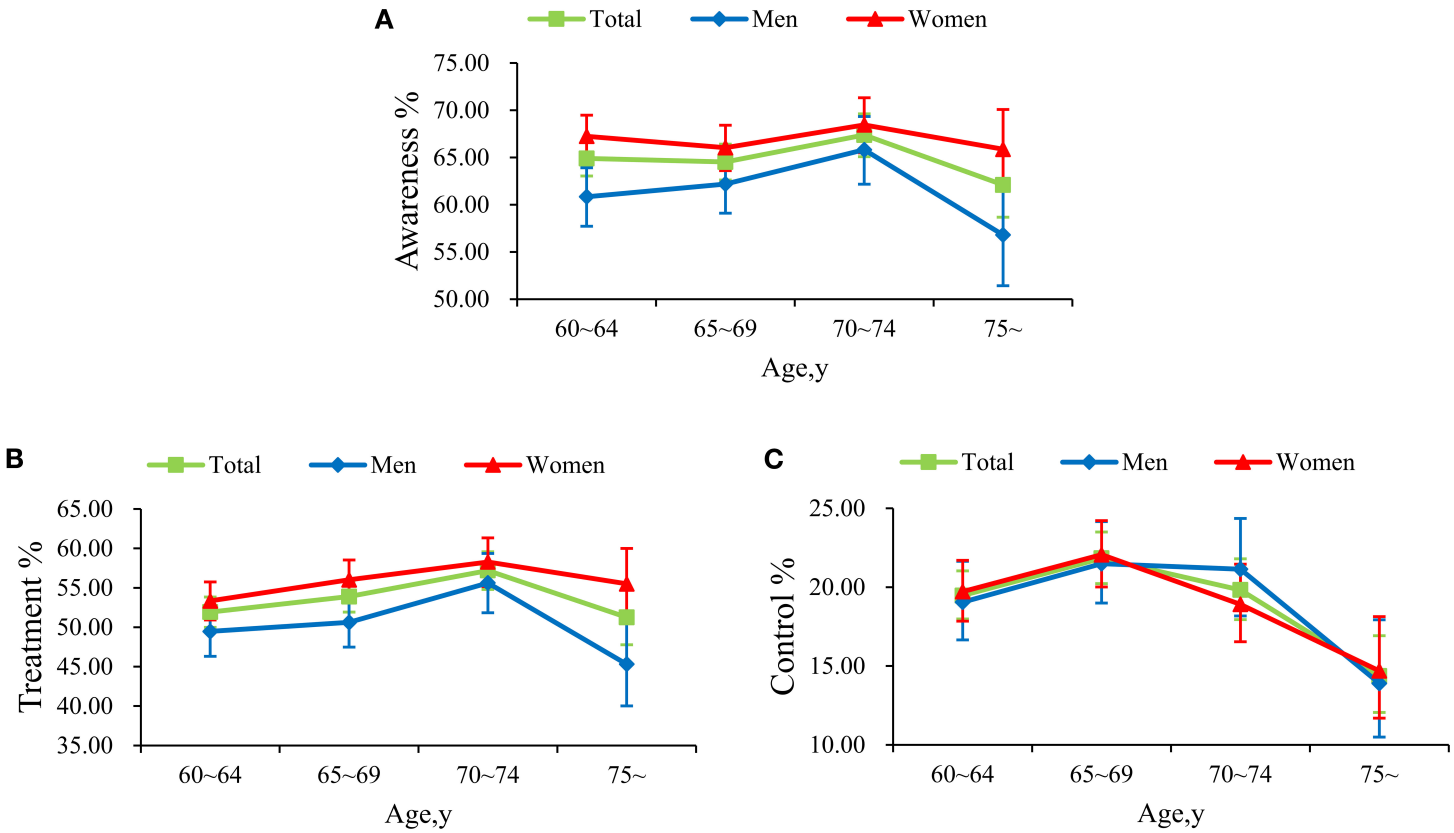
Awareness **(A)**, treatment **(B)**, and control **(C)** of hypertension between various genders.

The awareness, treatment, and control of hypertension was 65.02%, 53.65%, and 19.78%, respectively. As was illustrated in Supplementary Table 2, the awareness and treatment of hypertension were significantly higher among women than men (*P* < 0.001), while no statistically significance in control of hypertension was found in men and women (*P* = 0.967). Moreover, the awareness and treatment of hypertension continued to rise in subjects aged over 65, but began to decrease sharply at 70 for both genders. Simultaneously, the control of hypertension increased from 60 years old but began to fall dramatically at 65, especially for women (Figure 2).

### Analyses of Potential Influencing Factors

Age, single/widowed/separated/divorced, overweight or obese, and family history of hypertension were positively related to hypertension (all ORs > 1, all *P* < 0.05), whereas, underweight, more vegetable and fruit intake, high fat diet, and current smoking had negative association (all ORs <1, all *P* < 0.05). What's more, those who were suffering from diabetes or dyslipidemia were more likely to have hypertension, whereas they also tended to be aware of treatment to control hypertension (all ORs > 1). More detailed descriptions concerning the potential risk factors of the awareness, treatment, and control of hypertension are revealed in Supplementary Figure 1.

### Evaluation of Health-Adjusted Life Expectancy

The life expectancy (LE) decreased with increasing age among participants in both genders, and the similar trend was also observed for the health-adjusted life expectancy (HALE) in the counterparts with or without hypertension. Across all the age groups in both genders, the HALE was higher among participants without hypertension compared with those with hypertension. For those aged 60 years, the LE was 22.0872 years (men, 20.3282 years; women, 23.9752 years) and the HALE was 15.5578 (men, 15.1060 years; women, 16.1084 years), and 15.9418 (men, 15.4428 years; women, 16.4581 years) for those with or without hypertension, respectively. Namely, in this particular age group, subjects without hypertension could gain 0.3840, 0.3368, and 0.3497 years of HALE for total, men, and women, respectively. Additionally, in the age group of 65, 70, and 75, the projected HALE was 0.3548, 0.3115, and 0.1927 years longer among participants without hypertension compared with their counterparts with hypertension. More details of LE and the HALE for men and women subgroups across age groups are displayed in Table 3.

**Table 3:** The life expectancy and health adjusted life expectancy of participants across various age groups.

**Age group (years)**		**Total LE^#^ (years)**	**HALE^&^ (years)**		
			**Without HTN**	**With HTN**	Difference^*^
Total					
	60~64	22.0872	15.9418	15.5578	0.3840
	65~69	18.1549	11.9173	11.5625	0.3548
	70~74	14.5802	8.0844	7.7729	0.3115
	75~	11.3126	4.2231	4.0304	0.1927
Men					
	60~64	20.3282	15.4428	15.1060	0.3368
	65~69	16.6262	11.5932	11.2865	0.3067
	70~74	13.3108	7.9327	7.6645	0.2682
	75~	10.2554	4.1685	3.9820	0.1865
Women					
	60~64	23.9752	16.4581	16.1084	0.3497
	65~69	19.7475	12.2307	11.9081	0.3226
	70~74	15.8435	8.2127	7.9250	0.2876
	75~	12.2877	4.2667	4.0832	0.1835

*HTN, hypertension; LE, life expectancy; HALE, health adjusted life expectancy.*

* *Gain in health adjusted life expectancy after eliminating hypertension.*

# *Total life expectancy = life expectancy in healthy status + life expectancy in unhealthy status.*

& *life expectancy in healthy status*.

## Discussion

The crude and age-standardized prevalence, awareness, treatment, and control of hypertension were 44.52%, 65.02%, 53.65%, 19.78% and 45.36%, 65.05%, 53.74%, 19.68%, respectively. The prevalence of hypertension was lower than 53.24% reported in a national study conducted in 2012–2015, and the awareness, treatment, and control rates were slightly higher than that national level of the older population (19). The LE and HALE were decreased with increasing age among participants in both genders, which was in line with other studies. Furthermore, within people in the age of 60, 65, 70, and 75, they would gain 0.3840, 0.3548, 0.3115, 0.1927 years of HALE after eliminating hypertension, respectively, which revealed the impact of hypertension on health to be more realistic. Therefore, increased attention should be paid to older adults in rural China, and more reasonable strategies and measures should be urgently proposed and adopted. Additionally, the huge prehypertension population (29.83%) in this study suggested a remarkable risk burden of cardiovascular and cerebrovascular diseases among older adults in rural regions. Investigators of Framingham Heart Study have reported that individuals with prehypertension were two-fold to three-fold more likely to progress to hypertension compared with those with normal BP (20).

The current study also demonstrated that hypertension was more common among old female than their male counterparts, which was in line with previous researches (15, 21). This phenomenon may be explained by the hormonal changes at different ages for men and women (15). It has been previously reported that the prevalence of hypertension in postmenopausal women was higher than in premenopausal ones (22). It is worth noting that the prevalence of hypertension kept increasing with age, whereas, as for prehypertension, the prevalence was maintained decreasing with age among participants, with the exception of the age group of 75–79. The findings might be because prehypertension, a phase in the progression to hypertension from normal BP, tends to be more severe over time and was inclined to progress to hypertension with aging (23). Another cohort study also reported that prehypertension tends to become severe over time (24). Simultaneously, the increasing trend of prehypertension that began at age 75 remained unexplainable in our study and needs to be elucidated by further studies. Concerning participants in the age group of 60, those without hypertension can live 0.3840 years more (0.3368 and 0.3497 years for men and women, respectively) in healthy status, compared with their counterparts suffering from hypertension. With the exception of those aged over 75, the difference between the gap of HALE in nonhypertensive and hypertensive groups was higher among women than men. In addition, the LE and HALE decreased sharply with increasing age among both genders, showing that the quality of life of rural older population deteriorated with age, which has been reported in other areas (25).

As was published in other epidemiologic studies conducted at home and abroad, age was associated positively with hypertension (26, 27). Simultaneously, what was displayed in logistic regression analysis was that the ORs displayed a significant increase with increasing age for them, which hinted that more attention should be paid to older people. In addition, being overweight and obese is associated with a higher risk of hypertension, as has been found in a great many investigations (5, 28). An obvious association between BP increase and weight gain was discovered (28–30), and a dose-response effect of the magnitude of weight loss on BP reduction was reported by the American obesity guideline (31). For overweight and obese individuals, hence, several lines of evidence suggest that weight loss strategy is of utmost importance to refrain from higher blood pressure readings (29). Demonstrated in numerous observational and intervention studies, more vegetable and fruit intake offered a considerable benefit against the risk of hypertension (32), and this phenomenon was also found in this study. Additionally, those suffering from diabetes or dyslipidemia were more inclined to develop hypertension, which was consistent with studies published previously (33, 34). Yin Ruixing et al., discovered that dyslipidemia was associated with hypertension in many aspects, and this excited several common risk factors among these chronic noncommunicable diseases (35). More attention ought to be paid to those with diabetes and dyslipidemia when screening for hypertesnion. Futhermore, the results suggested that moderate or high physical activity was related to lower risk of having hypertension, in line with other publications. Given what was reported in a review from 27 randomized controlled trials, medium-to-high-intensity aerobic activity would reduce the BP by 11/5 mmHg among hypertensives (36). Therefore, for people with hypertension it would better to exercise more to lower BP.

The strengths of the study should be highlighted. Firstly, this was the latest study to explore the status of hypertension and health-adjusted life expectancy focusing on older population in rural regions. In addition, the present study covered a large sample of study population, which made the results more convincing. Nevertheless, several limitations of this study should be noted as well. Firstly, due to the crosssectional nature, the HALE was calculated by Sullivan method with death data from the 2017 China Cause-of-death Surveillance Data set. As the LE and HALE were calculated with crosssectional data, the LE and HALE in our study may be relatively underestimated. Indeed, follow-up surveys of this cohort are still ongoing and subsequent data would make the HALE more convincing by evaluating *via* multistate life table method. Secondly, considering all participants contained in the present study were older adults from rural areas in China, the generalizability of the current reported information to other regions or populations could be limited. Finally, the information on lifestyles and personal history of diseases was collected *via* a standardized questionnaire, as a consequence, recall bias was unavoidable. However, the questionnaire was concerned with high reliability and validity, and the researchers had been well-trained, and so the results in the present study were convincing even if there exsited recall bias.

In conclusion, the prevalence of hypertension was relatively high while the awareness, treatment, and control were fairly low. The HALE of older adults in resource-limited areas could increase from the reduction of hypertension. Unhealthy lifestyles should be adjusted and relevant policies should be formulated to help the older ones in rural areas access to reduce hypertension prevalence and increase the health-adjusted life expectancy for this population.

## Data Availability Statement

The raw data supporting the conclusions of this article will be made available by the authors, without undue reservation.

## Ethics Statement

The study was approved by Zhengzhou University Life Science Ethics Committee (Code: [2015] MEC (S128)) and conducted following the principles of the Declaration of Helsinki. Additionally, written informed consent was obtained from each participant.

## Author Contributions

ZH: carried out data analysis and wrote the manuscript. XL, WL, NK, LM, ZM, JH, and WH: recruited the participants and administered the assessment. YL and CW: designed this study and guided the writing. All authors listed have read, corrected, and approved the final manuscript.

## Funding

This research was supported by the Philosophy and Social Science Planning Project of Henan Province (Grant No: 2020BSH018), Foundation of National Key Program of Research and Development of China (Grant No: 2016YFC0900803), Science and Technology Innovation Team Support Plan of Colleges and Universities in Henan Province (Grant No: 21IRTSTHN029), National Natural Science Foundation of China (Grant Nos: 81602925 and 82003543), Foundation of Medical Science and Technology of Henan province (Grant Nos: 201702367 and 2017T02098), and Discipline Key Research and Development Program of Zhengzhou University (Grant Nos: XKZDQY202008 and XKZDQY202002). The funders had no role in the study design, data collection and analysis, decision to publish, or preparation of the manuscript.

## Conflict of Interest

The authors declare that the research was conducted in the absence of any commercial or financial relationships that could be construed as a potential conflict of interest.

## Publisher's Note

All claims expressed in this article are solely those of the authors and do not necessarily represent those of their affiliated organizations, or those of the publisher, the editors and the reviewers. Any product that may be evaluated in this article, or claim that may be made by its manufacturer, is not guaranteed or endorsed by the publisher.

## Abbreviations

DBP: Diastolic blood pressure
SBP: Systolic blood pressure
BMI: Body mass index
IPAQ: Physical Activity Questionnaire
BP: Blood pressure
OR: Odds ratio
PTN: Prehypertension
NTN: Normotensive
HTN: Hypertension
PP: Pulse pressure
SD: Standard deviation
LE: Life expectancy
HALE: Health adjusted life expectancy
EQ-5D: EuroqOL-5D.
