# Supplementary material for: Prevalence and Health-Adjusted Life Expectancy Among Older Adults With Hypertension in Chinese Rural Areas
Source: Front Public Health. 2022 Mar 1;10:802195. doi: 10.3389/fpubh.2022.802195 (PMC8921077; doi:10.3389/fpubh.2022.802195)
Supplement: Supplementary file 1 [file Data_Sheet_1.docx]

**Supplementary appendix**

**Supplement to:** Prevalence and health adjusted life expectancy among the elderly with hypertension in Chinese rural areas.

**The flow chart for participant inclusion/exclusion**

**
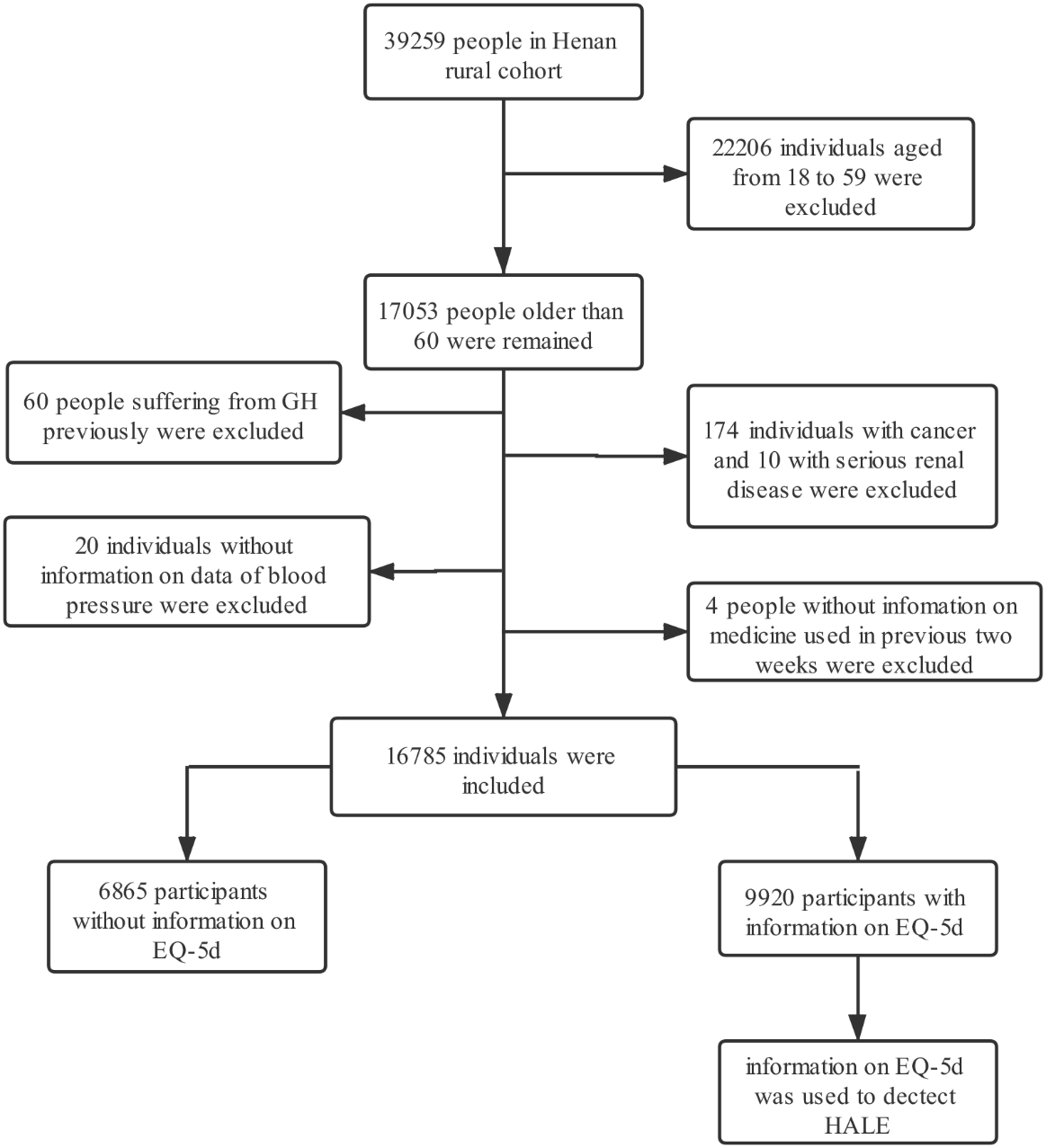
**

**The calculation of health adjusted life expectancy**

The respondents objectively evaluated their current health status via a Chinese version of the EQ-5D-5L instrument with the help of trained investigators. The utility values of EQ-5D index were derived by applying the preference weights of the general Chinese population sample via the time trade-off method ^[1]^. The index values require valuations for all relevant health states on a scale anchored at 1 (full health) and 0 (dead) ^[2]^. The number of population and deaths derived from 2017 China Cause-of-death Surveillance Data set was used to compile abridged life tables using the Chiang’s method ^[3]^ to obtain the estimate of LE. The HALE was further estimated via Sullivan method combining with LE and utility values of EQ-5D by the following formula ^[4, 5]^:

$$HALE=\frac{1}{l_{x}}\sum_{x=0}^{w} {}_{n}h_{x}\times{}_{n}L_{x}$$

where $l_{\begin{matrix} x \\ \end{matrix}}$ is the number of survivors at the exact age x; ${}_{n}h_{x}$represents the prevalence of a determined state of health among individuals with age in the interval (x, x + n), namely the average of utility values in the age interval in this study; ${}_{n}L_{x}$ is the number of person years lived in each age interval; and $w$ represents the maximum age.

**The IPAQ and the cutoffs used for low, moderate and high activity are as follows:**

| **Cutoff points** | **The IPAQ (MET-minute/week)** |
| --- | --- |
| low | <600 |
| moderate | 600~3000 |
| high | >3000 |

Abbreviations: IPAQ, International Physical Activity Questionnaire; MET, Metabolic equivalent of task

**The assertion of diabetes and dyslipidemia**

Diabetes was defined as fasting plasma glucose (FPG) ≥ 7.0 mmol/L or self-reported previously diagnosed by physicians and taking hypoglycemic drugs during the previous 2 weeks ^[6]^. According to the Chinese guidelines on prevention and treatment of dyslipidemia in adults ^[7]^, the cut-off values for high total cholesterol (TC), high triglyceride (TG), low high-density lipoprotein cholesterol (HDL-C) and high low-density lipoprotein cholesterol (LDL-C) were 6.22 mmol/L (240 mg/dL), 2.26 mmol/L (200 mg/dL), 1.04 mmol/L (40 mg/dL), and 4.14 mmol/L (160 mg/dL), respectively. Dyslipidemia was defined as the presence of one or more abnormal serum lipid concentrations or use of anti-dyslipidemia medications in the past two weeks.

**Table Legends**

**Supplementary Table 1.** Reported health problems of respondents.

**Supplementary Table 2.** The awareness, treatment and control of hypertension of total participants.

**Figure Legends**

**Supplementary Figure 1.** Multivariable adjusted odds ratios for the prevalence (A), awareness (B), treatment (C) and control (D) of hypertension.

**Supplementary Table 1.** Reported health problems of respondents.

| **Variables** | **Normotension** | **Prehypertension** | **Hypertension** | **P** |
| --- | --- | --- | --- | --- |
|  | **(N=2317)** | **(N=2857)** | **(N=4746)** |  |
| **Mobility (n, %)** |  |  |  | <0.001 |
| No problems | 1950 (84.16) | 2345 (82.08) | 3548 (74.76) |  |
| Slight problems | 275 (11.87) | 372 (13.02) | 801 (16.88) |  |
| Moderate problems | 63 (2.72) | 107 (3.75) | 273 (5.75) |  |
| Severe problems | 29 (1.25) | 30 (1.05) | 98 (2.06) |  |
| Extreme problems | 0 (0.00) | 3 (0.11) | 26 (0.55) |  |
| **Self-care (n, %)** |  |  |  | <0.001 |
| No problems | 2214 (95.55) | 2703 (94.61) | 4367 (92.01) |  |
| Slight problems | 68 (2.93) | 101 (3.54) | 234 (4.93) |  |
| Moderate problems | 21 (0.91) | 34 (1.19) | 89 (1.88) |  |
| Severe problems | 14 (0.60) | 17 (0.60) | 36 (0.76) |  |
| Extreme problems | 0 (0.00) | 2 (0.07) | 20 (0.42) |  |
| **Usual activities (n, %)** |  |  |  | <0.001 |
| No problems | 2138 (92.27) | 2604 (91.14) | 4110 (86.60) |  |
| Slight problems | 130 (5.61) | 173 (6.06) | 396 (8.34) |  |
| Moderate problems | 34 (1.47) | 50 (1.75) | 144 (3.03) |  |
| Severe problems | 14 (0.60) | 23 (0.81) | 59 (1.24) |  |
| Extreme problems | 1 (0.04) | 7 (0.25) | 37 (0.78) |  |
| **Pain/discomfort (n, %)** |  |  |  | 0.074 |
| No problems | 1689 (72.90) | 2091 (73.19) | 3375 (71.11) |  |
| Slight problems | 460 (19.85) | 572 (20.02) | 1013 (21.34) |  |
| Moderate problems | 119 (5.14) | 153 (5.36) | 273 (5.75) |  |
| Severe problems | 47 (2.03) | 38 (1.33) | 71 (1.50) |  |
| Extreme problems | 2 (0.09) | 3 (0.11) | 14 (0.29) |  |
| **Anxiety/depression (n, %)** |  |  |  | 0.04 |
| No problems | 2120 (91.50) | 2683 (93.91) | 4378 (92.25) |  |
| Slight problems | 132 (5.70) | 118 (4.13) | 247 (5.20) |  |
| Moderate problems | 44 (1.90) | 41 (1.44) | 87 (1.83) |  |
| Severe problems | 20 (0.86) | 11 (0.39) | 28 (0.59) |  |
| Extreme problems | 1 (0.04) | 4 (0.14) | 6 (0.13) |  |

**Supplementary Table 2.** The awareness, treatment and control of hypertension of total participants.

| **Variable** | **Awareness, n (%)** | ***P*** | **Treatment, n (%)** | ***P*** | **Control, n(%)** | ***P*** |
| --- | --- | --- | --- | --- | --- | --- |
| **Gender** |  | <0.001 |  | <0.001 |  | 0.962 |
| Men | 1807 (61.97) |  | 1481 (50.79) |  | 576 (19.75) |  |
| Women | 3051 (66.97) |  | 2528 (55.49) |  | 902 (19.80) |  |
| **Marital status** |  | 0.186 |  | 0.083 |  | 0.500 |
| Married/cohabiting | 3916 (64.66) |  | 3220 (53.17) |  | 1207 (19.93) |  |
| Widowed/single/divorced/separation | 942 (66.53) |  | 789 (55.72) |  | 271 (19.14) |  |
| **Education^*^** |  | 0.716 |  | 0.857 |  | 0.008 |
| Illiterate | 1561 (64.93) |  | 1280 (53.24) |  | 439 (18.26) |  |
| Primary school | 1886 (64.59) |  | 1577 (54.01) |  | 569 (19.49) |  |
| Middle school and above | 1411 (65.69) |  | 1152 (53.63) |  | 470 (21.88) |  |
| **Per capita monthly income (RMB)** |  | 0.007 |  | 0.049 |  | 0.022 |
| <500 | 2345 (65.98) |  | 1950 (54.87) |  | 738 (20.77) |  |
| 500~ | 1462 (62.48) |  | 1208 (51.62) |  | 419 (17.91) |  |
| 1000~ | 1051 (66.60) |  | 851 (53.93) |  | 321 (20.34) |  |
| **Body mass index (kg/m^2^)** |  | <0.001 |  | <0.001 |  | 0.022 |
| Underweight | 1427 (57.42) |  | 1115 (44.87) |  | 453 (18.23) |  |
| Normal | 68 (55.74) |  | 51 (41.80) |  | 22 (18.03) |  |
| Overweight | 2110 (67.01) |  | 1755 (55.73) |  | 625 (19.85) |  |
| Obese | 1218 (72.89) |  | 1056 (63.20) |  | 369 (22.08) |  |
| **High fat diet** | 485 (60.47) | 0.004 | 383 (47.76) | <0.001 | 149 (18.58) | 0.366 |
| **More vegetable and fruit intake** | 1637 (66.28) | 0.111 | 1322 (53.52) | 0.866 | 534 (21.62) | 0.005 |
| **Smoking** |  | <0.001 |  | <0.001 |  | 0.149 |
| Never | 3662 (65.86) |  | 3035 (54.59) |  | 1094 (19.68) |  |
| Former | 605 (66.27) |  | 505 (55.31) |  | 200 (21.91) |  |
| Current | 591 (59.16) |  | 469 (46.95) |  | 184 (18.42) |  |
| **Drinking** |  | <0.001 |  | <0.001 |  | <0.001 |
| Never | 3965 (65.72) |  | 3297 (54.65) |  | 1211 (20.07) |  |
| Former | 353 (73.39) |  | 304 (63.20) |  | 120 (24.95) |  |
| Current | 540 (56.37) |  | 408 (42.59) |  | 147 (15.34) |  |
| **Physical activity** |  | <0.001 |  | <0.001 |  | <0.001 |
| Low | 2160 (68.07) |  | 1862 (58.68) |  | 677 (21.34) |  |
| Moderate | 1540 (66.24) |  | 1249 (53.72) |  | 477 (20.52) |  |
| High | 1158 (58.66) |  | 898 (45.49) |  | 324 (16.41) |  |
| **Family history of hypertension** | 1264 (86.28) | <0.001 | 1110 (75.77) | <0.001 | 416 (28.40) | <0.001 |
| **Dyslipidemia** | 2429 (70.90) | <0.001 | 2079 (60.68) | <0.001 | 770 (22.48) | 0.375 |
| **Diabetes** | 901 (73.01) | <0.001 | 804 (65.15) | <0.001 | 304 (24.64) | <0.001 |

* In our study, primary school represents six schooling years, and middle school represents three more years based on primary school. And we added the schooling years in revised manuscript.


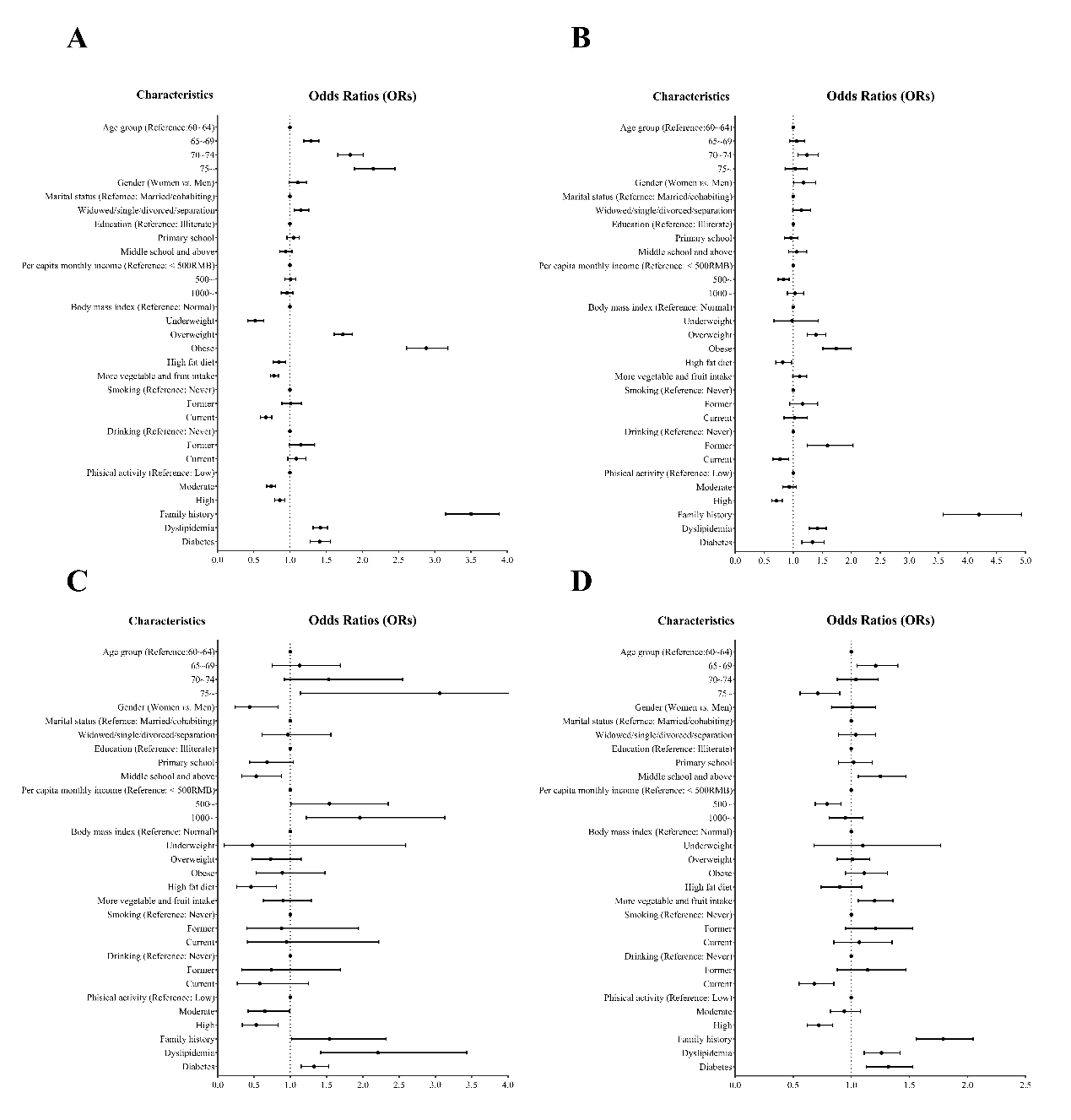


**Supplementary Figure 1.** Multivariable adjusted odds ratios for the prevalence (A), awareness (B), treatment (C) and control (D) of hypertension.

**References**

[1] N. Luo, G. Liu, M. Li *et al.* Estimating an EQ-5D-5L Value Set for China[J]. Value Health, 2017, 20(4): 662-669. 10.1016/j.jval.2016.11.016.

[2] T. Zhang, W. Shi, Z. Huang *et al.* Gender and ethnic health disparities among the elderly in rural Guangxi, China: estimating quality-adjusted life expectancy[J]. Glob Health Action, 2016, 9: 32261. 10.3402/gha.v9.32261.

[3] C.L. Chiang. Introduction to stochastic processes in biostatistics[J]. Wiley, 1968:

[4] D. Lim, J. Bahk, M. Ock *et al.* Income-related inequality in quality-adjusted life expectancy in Korea at the national and district levels[J]. Health Qual Life Outcomes, 2020, 18(1): 45. 10.1186/s12955-020-01302-6.

[5] B. Collins. Results from a Well-Being Survey in the North West of England: Inequalities in EQ-5D-Derived Quality-Adjusted Life Expectancy Are Mainly Driven by Pain and Mental Health[J]. Value Health, 2017, 20(1): 174-177. 10.1016/j.jval.2016.08.004.

[6] Y. Xu, L. Wang, J. He *et al.* Prevalence and control of diabetes in Chinese adults[J]. JAMA, 2013, 310(9): 948-959. 10.1001/jama.2013.168118.

[7] X. Liu, S. Yu, Z. Mao *et al.* Dyslipidemia prevalence, awareness, treatment, control, and risk factors in Chinese rural population: the Henan rural cohort study[J]. Lipids Health Dis, 2018, 17(1): 119. 10.1186/s12944-018-0768-7.
